# DEVICE-MEASURED DAILY PHYSICAL ACTIVITY IN OLDER ADULTS WITH DEMENTIA: CROSS-SECTIONAL RESULTS FROM THE HUNT STUDY

**DOI:** 10.1093/geroni/igae098.2271

**Published:** 2024-12-31

**Authors:** Karen Sverdrup, Paul Jarle Mork, Bjørn Heine Strand, Linda Ernstsen, Gro Gujord Tangen

**Affiliations:** The Norwegian National Centre for Ageing and Health, Tønsberg, Oslo, Norway; Norwegian University of Science and Technology, Trondheim, Sor-Trondelag, Norway; Norwegian Institute of Public Health, Oslo, Oslo, Norway; Norwegian University of Science and Technology, Trondheim, Sor-Trondelag, Norway; Sykehuset i Vestfold HF (Nasjonalt senter for aldring og helse), Tønsberg, Oslo, Norway

## Abstract

To provide accurate estimations of physical activity (PA) in epidemiological research and population studies, the use of accelerometers has skyrocketed over the past decade. However, implementation of accelerometers to accurately estimate PA in people with dementia is lagging. This population-based, cross-sectional study aimed to quantify daily PA in people with dementia categorised by sex and dementia subtype, using data from the ≥70 years cohort in the fourth wave of the Norwegian Trøndelag Health Study (HUNT4 70+). Clinical experts diagnosed dementia and subtypes according to DSM-5 criteria. PA was measured with two tri-axial accelerometers on the lower back and thigh for seven consecutive days. PA types and postures were classified using a validated machine learning model and analysed as mean minutes per day (min/d). Moderate-to-vigorous PA (MVPA) was defined as walking, running, and cycling, and analysed as mean minutes per week (min/w). Of the 1894 participants diagnosed with dementia, 577 participants had valid PA data. These participants spent 231 min/d in total PA, most of which was in standing (187 min/d). Mean time in MVPA was 50 min/w and only 4.3% met PA recommendations (≥150min/w). Men spent more time in MVPA than women (p< 0.001), whereas women spent more time standing (p=0.008). There were no differences between dementia subtypes in volume or intensity of PA. These results indicate that, in people with dementia, independent of dementia subtype and sex, there exists an enormous disadvantageous gap between performed PA and recommended levels of PA.

